# MALDI-ToF Mass Spectrometry for the Rapid Diagnosis of Cancerous Lung Nodules

**DOI:** 10.1371/journal.pone.0097511

**Published:** 2014-05-15

**Authors:** Fabienne Brégeon, Geoffrey Brioude, Florence De Dominicis, Thérèse Atieh, Xavier Benoit D'Journo, Christophe Flaudrops, Jean-Marc Rolain, Didier Raoult, Pascal Alexandre Thomas

**Affiliations:** 1 Aix-Marseille Université, Unité de Recherche sur les Maladies Infectieuses et Tropicales Émergentes (URMITE) UMR 63 CNRS 7278 IRD 3R198 INSERM U1095, IHU Méditerranée Infection, Facultés de Médecine et de Pharmacie, Marseille, France; 2 Explorations Fonctionnelles Respiratoires Centre Hospitalo-Universitaire Nord, Pôle thoracique et cardio-vasculaire, Assistance Publique Hôpitaux de Marseille, Marseille, France; 3 Service de chirurgie thoracique et des maladies de l'œsophage, Pôle thoracique et cardio-vasculaire, Centre Hospitalo-Universitaire Nord, Assistance publique-Hôpitaux de Marseille, Aix-Marseille université, Marseille, France; 4 Pôle des Maladies Infectieuses et Tropicales Clinique et Biologique, Fédération de Bactériologie-Hygiène-Virologie, Centre Hospitalo-Universitaire Timone, Assistance publique des hôpitaux de Marseille, Marseille, France; Baylor College of Medicine, United States of America

## Abstract

Recently, tissue-based methods for proteomic analysis have been used in clinical research and appear reliable for digestive, brain, lymphomatous, and lung cancers classification. However simple, tissue-based methods that couple signal analysis to tissue imaging are time consuming. To assess the reliability of a method involving rapid tissue preparation and analysis to discriminate cancerous from non-cancerous tissues, we tested 141 lung cancer/non-tumor pairs and 8 unique lung cancer samples among the stored frozen samples of 138 patients operated on during 2012.

Samples were crushed in water, and 1.5 µl was spotted onto a steel target for analysis with the Microflex LT analyzer (Bruker Daltonics). Spectra were analyzed using ClinProTools software. A set of samples was used to generate a random classification model on the basis of a list of discriminant peaks sorted with the *k*-nearest neighbor genetic algorithm. The rest of the samples (n = 43 cancerous and n = 41 non-tumoral) was used to verify the classification capability and calculate the diagnostic performance indices relative to the histological diagnosis. The analysis found 53 m/z valid peaks, 40 of which were significantly different between cancerous and non-tumoral samples. The selected genetic algorithm model identified 20 potential peaks from the training set and had 98.81% recognition capability and 89.17% positive predictive value. In the blinded set, this method accurately discriminated the two classes with a sensitivity of 86.7% and a specificity of 95.1% for the cancer tissues and a sensitivity of 87.8% and a specificity of 95.3% for the non-tumor tissues. The second model generated to discriminate primary lung cancer from metastases was of lower quality.

The reliability of MALDI-ToF analysis coupled with a very simple lung preparation procedure appears promising and should be tested in the operating room on fresh samples coupled with the pathological examination.

## Introduction

Surgery is often the key element of treating tumoral masses, but the difficulty of determining an exact etiologic diagnosis prior to the surgery often leads to operations being performed without prior knowledge of precisely whether limited or extended resection is required, especially when the lesion is smaller than 5 mm in diameter. In some cases, such as brain tumors, the question of the resection margin increases the difficulty of the decision, and surgeons have to balance maximizing the resection of tumor and minimizing the potential for functional deficit in preserving critical tissue [1]. In other cases, such as emergency surgery, a mass of unknown origin may be revealed unexpectedly, thus raising the question of whether the tumor is of cancerous origin and requires extensive resection. Real-time confirmation methods are therefore required to guide the surgeon in tissue resection and to optimize treatment [2]. Confirmation usually relies on intraoperative pathologic examination of frozen sections that can provide information within an hour. In lung cancer surgery, frozen section diagnosis directly influences surgical decision making [3]: when malignancy is identified on a frozen section following a wedge resection, surgical resection by lobectomy or pneumonectomy is usually performed, as recommended by the American College of Chest Physicians [4]. Because frozen section analysis is typically limited and involves no cell labeling or staining, it can yield false positives and false negatives. It has been associated to more than 7% discordant or doubtful results in some studies [3], [5] and up to a 42% misclassifications rate in safety margin assessment in certain lung cancer studies [6]. In the absence of complementary methods for tissue analysis in the operating room, decisive action has to be taken before the definite diagnosis. Finally, definite diagnosis relies on standard histopathology based on cytology/nuclei abnormalities and is usually supplemented with the analysis of changes in genomics and transcriptomics.

Proteomics is used to study the large spectrum of genome-encoded proteins present at a given time [7]. Although the first use of mass spectrometry in cancerous disease was in the 2000s [8], this approach is complex, requiring time-consuming tissue or sample conditioning. Targeting the identification of specific biomarkers of cancers has led to disappointing results. Recently, matrix-assisted laser desorption ionization time-of-flight mass spectrometry (MALDI-ToF MS) has been applied to cryosectioned samples of tumoral tissue; the resulting spectra were combined with histological micro-imaging of the same section to classify tumors with acceptable accuracy [9]. This method of MALDI-imaging has the advantage of being conservative but would require expert analysis and delayed interpretation that is incompatible with the rapid responses needed by the surgeon and with the ability to use the method in an operating room. By contrast, high-throughput and rapid proteome spectra can be obtained from MALDI-ToF MS analysis of complex samples with minimal pretreatment, and this method has been shown to enable species classification of whole complex organisms including ticks [10]. It also allows bacteria identification in complex media, such as blood [11] and urine [12] without colony culture.

Hypothesizing that rapid MALDI-ToF MS analysis of a crude crushed tissue sample could be informative, the aim of the present pilot study was to evaluate the reliability of MALDI-ToF MS to rapidly classify a crude lung tissue sample of unknown origin as cancerous or non-cancerous using a minimal sample volume and a simple preparation method that could be performed in the operating room.

## Materials and Methods

### Study design

All samples were collected from lung surgical specimens from patients undergoing thoracic surgery for cancer (AP-HM, Hôpital Nord, Marseille) between January 2012 and December 2012. Written informed consent was obtained from all patients. The protocol was approved by the National Ethics Committee "Comité d'Ethique de la Recherche Clinique en Chirurgie Thoracique et Cardio-Vasculaire (CERC-CTCV) (reference number: CERC-SFCTCV-2012-1-31-11-35-32-DeFl).

### Sample collection

During the surgical procedure and immediately after lung resection, biopsies were taken from the resected specimens of non-tumoral (Non-tumor) and tumoral parts (Cancer) of the lungs. Sampling was performed without compromising the diagnostic quality of the piece designated for histological analysis and never required more tissue resection than that necessary for the therapeutic management of the patient. When the tumoral mass was apparently small in size, the entire tumoral piece was dedicated to the histological examination; thus, only tumor resections of more than 1 cm were included in the study. In those cases, a tissue specimen was reserved for the study, snap-frozen and stored at -80°C for further MALDI-ToF MS analysis. When there was enough material, it was subdivided in two sets of samples that were considered individually. Independently, the main tumor sample was sent for pathological examination, and patients were assigned a TNM postsurgical stage score according to the international lung cancer staging system. According to the standard WHO criteria [13], the cancers were classified histologically into adenocarcinoma, squamous-cell carcinoma, undifferentiated carcinoma, carcinoid carcinoma, lymphoma and sarcoma.

From the whole sample list, 2/3 were randomly assigned to a reference training set (Reference), which constituted a data base with an equal proportion of Non-tumor and Cancer samples. The remaining third and all the samples from atypical and/or extremely rare cancers were used to design a blinded group (Blinded). The Cancer and Non-tumor samples from the same patient were distributed randomly in either the Reference or Blinded pool.

### Preparation of Samples for Mass Spectrometry

At the time of the analysis, each frozen sample was thawed at room air for approximately 15 min., and cut with a sterile scalpel. Using a laboratory microbalance (CPA 224S, Sartorius Stedim Aubagne, France), 0.1 g (0.1±0.008 g) was placed in a 10 ml sterile glass tube added with 0.9 ml sterile water to obtain 10% dilution. When enough amount of tissue was available, a second piece was processed in order to perform tests in duplicate. The tissue was homogenized in water using IKA ULTRA-TURRA X T25 (IKA-Werke GmbH & CO. KG. Staufen, Germany) at 17000 rpm during 2 min.. The temperature was not maintained under control during the homogenization process. To obtain a 1/160 dilution, 100 µl of the mixture was taken and diluted again in 1.5 ml of sterile water and vortexed. No additional component, especially no inhibitor was added to the mixture during the whole process. 1.5 µl of this dilution was spotted in quadruplicate onto a 96-sample polished steel target. After drying on the bench, 1.5 µl of HCCA matrix was added for ionization. Air-dried targets were measured immediately. Each lung sample generated 4 spectra from the 4 deposits.

### Mass Spectrometry

Measurements were performed with a Microflex LT (Bruker Daltonics, Bremen, Germany) mass spectrometer laser. Spectra were recorded in the positive linear mode (delay: 170 ns; ion source 1 (IS1) voltage: 20 kV; ion source 2 (IS2) voltage: 16.65 kV; lens voltage: 7.20 kV; mass range: 2 kDa to 20 kDa). Each spectrum was obtained after 6×40 shots (240 shots) in automatic mode at a variable laser power, and the acquisition time ranged from 60–120 seconds per spot. All signals with resolution ≥ 400 were automatically acquired using AutoXecute acquisition control in FlexControl software version 3.0. The spectra of the 4 spots for each tissue mix were imported into BioTyper-RTC version 3.0 software (Bruker Daltonik GmbH).

### Statistical analysis

ClinProTools v2.2 software uses the data generated from spectra including spectra pretreatment, peak picking, and peak calculation operation. The peak definition, the normalization of the area to the total ion count end point level and the mass recalibration (maximal peak shift of 1000 ppm) were taken into account, and the sort mode using the t-test p-value from the Wilcoxon/Kruskal-Wallis test was used.

Differences in classes analyzed were assessed on the basis of a discriminant peak identification list. To create the list of discriminant peaks, we used the *k*-nearest neighbor genetic algorithm (GA) implemented in this software. This algorithm is based on probability estimates for classification.

We first searched for a model able to correctly discriminate the 2 classes, Cancer and Non-tumor, and second for a model able to correctly discriminate Primary lung cancer and Metastasis. To find the most discriminant model, GA was trained with the Reference pool, and internal validation was processed (10-fold cross-validation). The performance of the model was evaluated by recognition capability (RC) and positive predictive value (PPV): RC  =  TP/n where TP is the number of true positives (correctly classified) in a data set, n is the number of samples in the data set, and PPV  =  TP/(TP + FP) where FP is the number of false positives (misclassified).

In a second step, the spectra from the Blinded samples were used to verify the classification ability of the generated model. The effective Sensitivity, Specificity and Accuracy of a model were calculated from the results obtained for the Blinded samples versus the reference histological diagnosis as the Gold standard using standard formulas (Sensitivity  =  TP/TP + FN; Specificity  =  TN/TN + FP; Accuracy  =  TP + TN/n).

For the first and the second step, duplicate material was tested after the best fit GA model was selected

## Results

For the classification of Cancer and Non-tumor entities, 290 samples were analyzed corresponding to 138 patients. From this cohort, there were 141 Cancer/Non-tumor pairs and 8 unique Cancer pieces. Of the 290 resection pieces, 225 gave enough materiel to perform duplicate analysis. Concerning the 149 cancer pieces, the definite tumor classification was primary lung cancer for 132 samples (83 adenocarcinoma, 34 squamous cell carcinoma, 5 undifferentiated carcinoma, 5 carcinoid tumors, 1 small cell lung carcinoma, 2 lymphoma and 2 sarcoma), and 17 were metastases.

**Figure 1:**
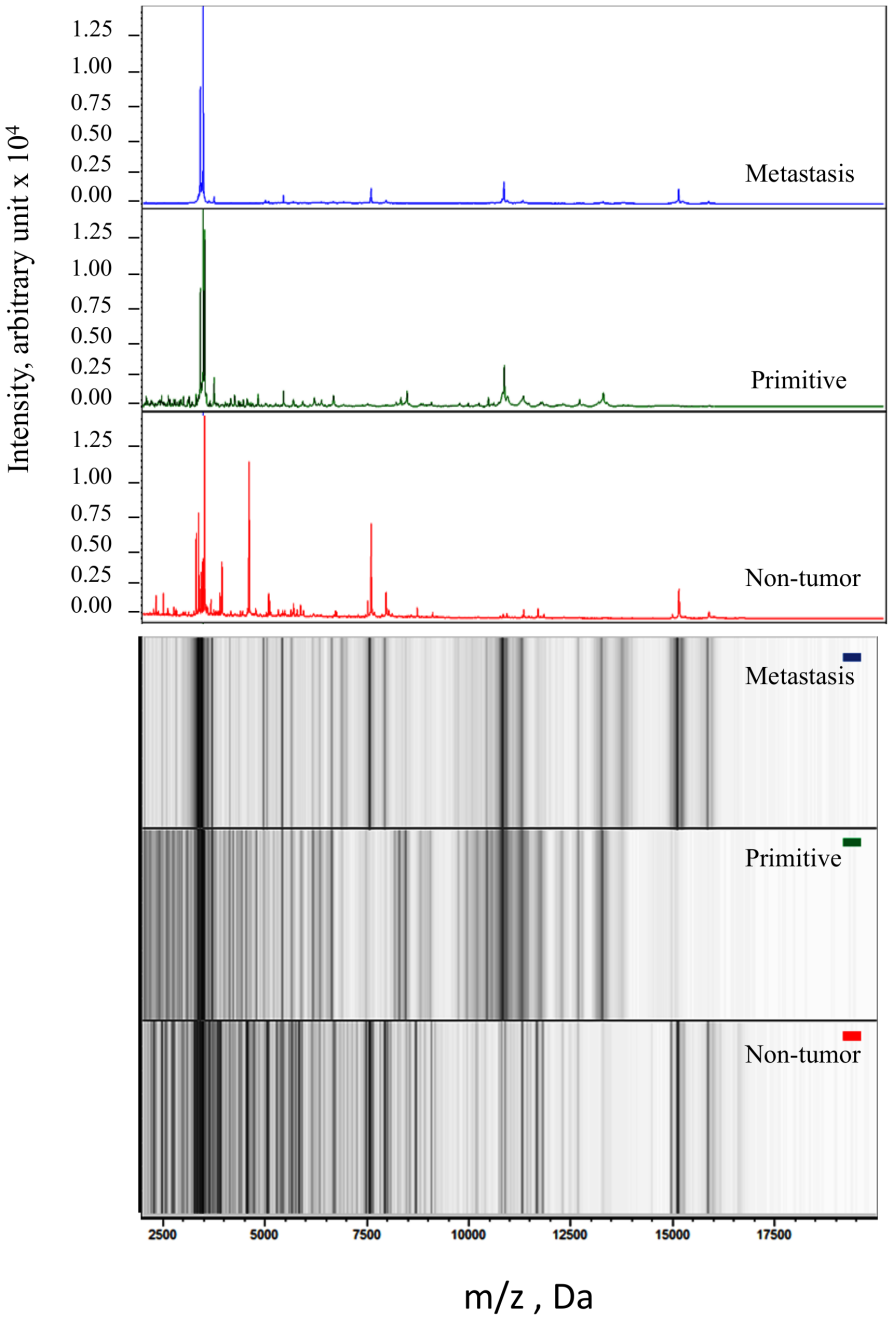
**top:** Representative spectra of each subclass: Non-tumor, Primary and Metastasis. **bottom:** Gel images in grayscale from the same samples as above.

**Figure 2:**
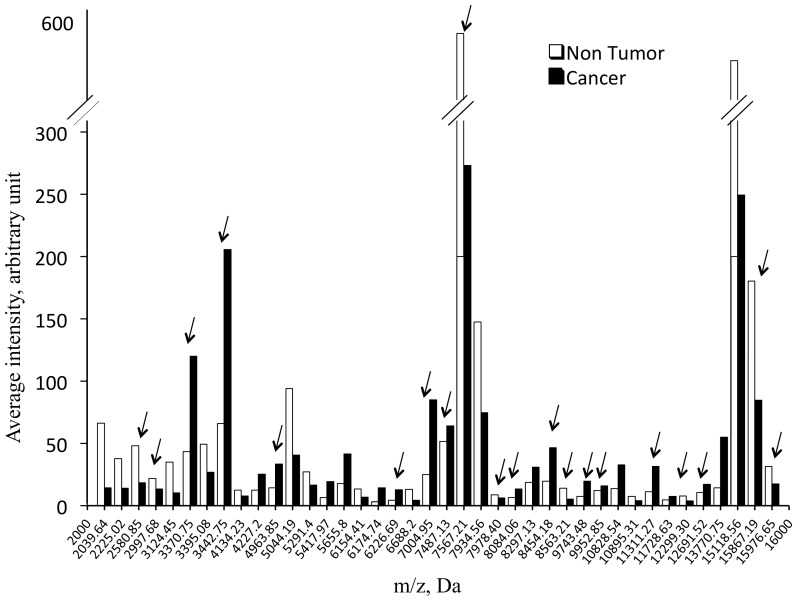
Average intensity versus mass-to-charge ratio of 40 significantly different peaks averaged from the whole cohort between Cancer and Non-tumor samples. Arrows show the mass values for the 20 peaks selected by the Cancer versus Non-tumor GA. The peaks #3370.75, 3442.75, 4963.85, 7004.95, 7487.13, 7567.21, 8454.18, 8563.21, and 9952.85 were up-regulated in the Cancer set, whereas the others were up-regulated in the Non-tumor set.

**Figure 3:**
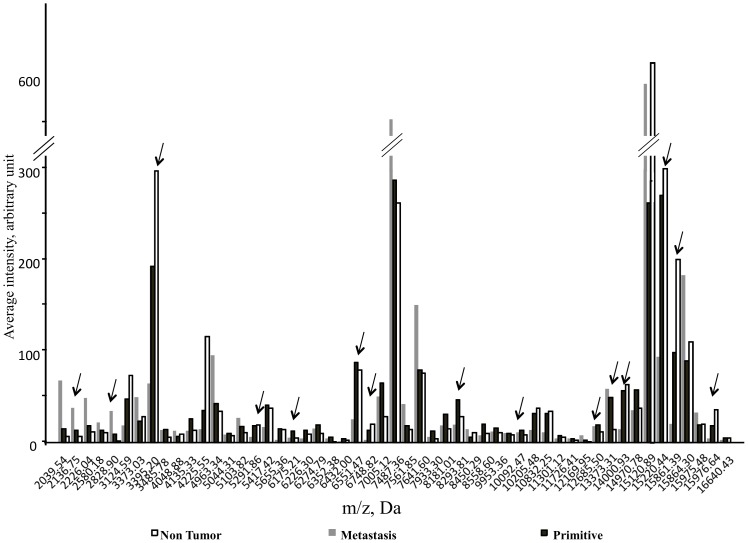
Average intensity versus mass-to-charge ratio of 49 peaks significantly different between Non-tumor, Primary and Metastasis subclasses averaged from the whole cohort. Arrows show the mass values for the 15 discriminating peaks selected by the Primary cancer versus Metastasis GA model. The peaks 2136.75, 2829.90, 5291.86, 6175.21, 6551.37, 6748.52, 8181.01, 10092.47 and 12685.50 were up-regulated in the Primary Cancer set, whereas the others were up-regulated in the Metastasis set.

Representative spectra from a Primary lung cancer (SCLC) sample, a Metastasis and a Non-tumor sample are shown in Figure 1. A total of 53 m/z peaks generated from Cancer and Non-tumor samples from the whole cohort were considered valid, with 40 of them being significantly different between both classes (p<0.001); these peaks are reported in Figure 2. Concerning the Primary lung cancer, Metastasis and Non-tumor subclasses, a total of 53 peaks were identified, and 49 of them being significantly different (p<0.001). These peaks are reported in Figure 3.

### Statistical data analysis and the Cancer versus Non-tumor GA classification model

To distinguish Cancer from Non-tumor samples, when parameter KNN = 3, MNG = 1000, and Max peaks = 250, the GA model fit was RC = 98.81%, PPV = 89.17% (Table 1) with 20 potential peaks (m/z: 8084.06, 4963.85, 12299.3, 12691.52, 7993.95, 7004.95, 2580.85, 9952.85, 8454.18, 6226.69, 2997.68, 9743.9, 8563.21, 15976.65, 11311.27, 15867.19, 7487.13, 7567.21, 3370.75, 3442.75). Analysis of spectra from the Blinded set (n = 43 Cancer and n = 41 Non-tumor) accurately discriminated the two classes with a sensitivity of 86.7% and a specificity of 95.1% for the Cancer class and a sensitivity of 87.8% and a specificity of 95.3% for the Non-tumor class.

**Table 1:** Diagnostic performances of the 20 peaks of the two class (Cancer versus Non-tumor) GA model using Reference and Blind sets of lung samples.

Reference set (n = 206)			
	Cancer	Non-tumor	Overall
Recognition Capability	98.29%	99.34%	98.81%
Positive Predictive Value	89.83%	88.5%	89.17%

Blinded set (n = 84)			
	Cancer	Non-tumor	Overall
Accuracy	91.6%	92.3%	92%
Sensitivity	86.7%	87.8%	87.25%
Specificity	95.1%	95.3%	95.2%

RC: Recognition Capability, PPV: Predictive Positive Value; Se: Sensibility; Sp: Specificity. Accuracy  =  TP + TN/TP + FN + FP + TN.

RC and PPV were calculated by testing the training cohort (n = 206). Se and Sp were calculated by testing the Blinded cohort (n = 84 samples).

When present, the duplicates of this Blinded set were also tested: in all the cases they obtained the same class allocation as the first sample.

### The Primary lung cancer versus Metastasis GA model

To discriminate Primary lung cancer from Metastases, with parameter KNN = 3, MNG = 1000 and Max peaks = 250, the GA model fit was RC = 100%, PPV = 90.24% with 15 potential peaks (2136.75, 2829.90, 3485.98, 5291.86, 6175.21, 65551.37, 6748.52, 8181.01, 10092.47, 12685.50, 13767.60, 14000.93, 15220.44, 15861.39, 15976.64). Analysis of the spectra from the Blinded set (n = 48, 40 Primary and 8 Metastasis) accurately discriminated the two subclasses with a sensitivity of 67.5% and specificity of 75% for the Primary subclass, and a sensitivity of 50% and a specificity of 70% for the Metastasis subclass. The accuracy was, respectively, 68.75% and 66.7%.

## Discussion

Due to its possible impact on patient surgical treatment, the rapid analysis of a tissue sample is of particular importance when a patient with a suspect mass is operated upon, especially when the tumor's origin is unknown or when the nature of the safety margins is questioned. In this pilot study, using a simple preparation method and the algorithm for sample classification implemented in the MALDI-ToF analysis software, we obtained acceptable diagnostic performance to correctly classify a lung sample as cancerous or non-cancerous. Although limited, such information could be of great help for completing frozen section pathological diagnostics when a rapid answer is required.

Lung cancer is the leading cause of cancer-related mortality and the most frequently diagnosed cancer worldwide, with approximately 1.35 million new cases each year, among which 30000 are in France. More than 80% of lung cancers are non-small cell lung cancer (NSCLC), for which surgical resection remains the single most consistent and successful option to achieve a cure. Sometimes, a pulmonary nodule is revealed to be non-cancerous *a posteriori*, and therefore, the rapid identification of the malignant origin of a tumor-like tissue is of great importance. Our Thoracic Surgery Department performs approximately 350 lung resections and explores approximately 30 nodules of unknown origin by thoracoscopy or conventional surgery each year. In addition, our research laboratory includes a proteomic platform and is familiar with the affordable and easy-to-use bench top MALDI-ToF Mass spectrometer; thus, the conditions necessary to perform the present pilot study were met.

Previous encouraging results were obtained using MALDI-ToF MS analysis combined with purification methods [14]. Using intact cell suspensions directly spotted on the matrix and analyzed by MALDI-ToF MS, valid and reproducible spectra were obtained from malignant neoplasms of the oral cavity, and a statistical model was able to correctly classify a cancerous sample with a sensitivity of 100%, a specificity of 93%, and an overall accuracy of 96.5% [14]. These results, which are better but close to ours, were obtained using spectral patterns from a homogenous population of cell suspensions. Recently, non-homogenous tissue-based methods have been developed for proteomic and lipidomic analysis, and they appear to be reliable for tumor classification for digestive, brain, lymphomatous, and lung cancers [15]–[18]. Among these tissue-based methods, MALDI-imaging is now used by several teams for clinical research. However, the MALDI-imaging approach remains complex because it requires frozen tissue slice analysis results to co-register MALDI spectra imaging and morphology imaging. For human liver metastasis samples, this method allowed tumor classification into six common cancer types with a sensitivity varying from 54% to 88%, and a specificity varying from 90% to 98% depending on the malignant class [9]. To simplify the process, Lee and coauthors proposed performing MALDI-ToF MS for lipidomics analysis of preselected frozen section slices containing at least 70% malignant cells [18]. The resulting spectra were used to generate a model (support vector machine algorithm) that accurately classified normal lung tissues, lung tumor tissues, and primary NSCLC. Primary NSCLC was accurately discriminated from other types of lung tumors, and the three subclasses, adenocarcinoma, squamous-cell and large-cell carcinoma, were correctly discriminated and classified with a sensitivity and a specificity of 84% and 77%, respectively for adenocarcinoma versus squamous cell carcinoma [18]. The authors recorded no misclassified sample when comparing Primary NSCLC and other types of lung tumors, whereas in the present study, we found both false negatives and false positives when we compared Primary lung cancer versus Metastasis subclasses. The difference in our study sample size, with greater numbers of tumoral and non-tumoral samples (respectively 149 and 141) compared to the above-mentioned study (respectively 47 and 6), could explain differences in diagnostic performance results. In addition, good diagnostic performance from other studies was achieved by applying MALDI-imaging on chosen regions that contained high tumor cellularity [1], [18] based on the histology of sections stained with hematoxylin and eosin. Here, we used no pre-selection of tissue samples and obtained good results. We targeted tumoral pieces larger than 1 cm which represent the most frequent surgical indications. It is plausible that the size of the tumor have favorably influenced our results since the risk of having sampled a bad territory is reduced with large tumors as compared to millimeter tumors.

Mass spectrometry imaging strategies offer the advantage of conserving tissue but require sufficient surface area of tissue sections to obtain valuable information. In addition, MS imaging methods require trained experts, heavy analysis software and high-throughput signal acquisition instrumentation. Like these above-mentioned methods, our strategy did not require any purification or standardization of the tissue cell content. Our crushed sample MS analysis was rapid, reproducible and very easy to perform. The non-conservative aspect of our approach was in part counterbalanced by the very low tissue sample size (i.e. approximately 0.01 g) able to give valid spectra. Finally, using a simplified and non-image-guided method and larger cohort of patients, we obtained diagnostic performances similar to those obtained with MALDI-imaging methods or purified cell line methods. This surprising result could be due to more the complete information contained in complete unpurified tissue sample and to our modest objective, which was not to identify the exact nature of the tumor but to classify the sample into either the Cancer or non-tumor class. Very interestingly, among the potential peaks that were selected in our GA model, three, i. e. 4963.85-8563.21-9952.85 were also highlighted in a study by Raham and coauthors who used extraction and purification methods and a GA model [19]. In addition, these authors identified the corresponding candidate proteins (Thymosin Ubiquitin and Acyl-coA binding protein) and confirmed their presence in the lung tumors by immunochemistry.

Microflex LT (Bruker Daltonics, Bremen, Germany) mass spectrometer laser is a bench disposable material with integrated analysis software that can be easily installed in the operating facilities. The novelty here is that the complete sample treatment process, including tissue dispersion, sample material deposition on the matrix and analysis, does not require technical expertise and could be learned by any paramedical personnel.

We used two third of our samples for building the prediction model whereas equal or lower numbers are commonly used for training sets compared to validation sets. This was justified by the heterogeneity of our Cancer population with the aim to increment the training set to obtain a large representation of reference cancerous spectra. Finally, our Blinded set population size was higher than previously published with MALDI-ToF MS on lung tissue (n = 84). In contrast with our good diagnostic performance in classifying a sample as Cancer versus Non-tumor, we obtained low performances for the Primary versus Metastasis subclasses. We think that the large diversity in metastasis subgroups contrasting with the low number of samples analyzed in this subclass could be responsible for a low performance random mathematical model. We hope that incrementing the training cohort with Metastasis would lead to finding a GA model with better diagnostic performance. Adopting complementary and/or alternative exatraction/solubilization methods would improve the yield of detecting m/z peaks. However, increasing preparation step should be balanced with regard to the application of this tool in clinical settings. At this stage of the work, we think it could be possible to give a result in less than 30 minutes, thus determining whether a sample is cancerous or not with a simplified and rapid approach for whole proteomic tissue analysis that could be easily used as a diagnostic aid during routine surgical procedures. The ability to have information reliably confirmed on-theater versus using frozen biopsies could have major implications for the management of patients with tumors.
